# Human haematopoietic stem cells express Oct4 pseudogenes and lack the ability to initiate Oct4 promoter-driven gene expression

**DOI:** 10.1186/1477-5751-9-2

**Published:** 2010-03-31

**Authors:** Zoe Redshaw, Alastair J Strain

**Affiliations:** 1 School of Veterinary Medicine and Science, The University of Nottingham, Sutton Bonington Campus Sutton Bonington, Leicestershire, LE12 5RD, UK; 2 School of Biosciences, The University of Birmingham, Edgbaston Birmingham, B15 2TT, UK

## Abstract

The transcription factor Oct4 is well defined as a key regulator of embryonic stem (ES) cell pluripotency. In recent years, the role of Oct4 has purportedly extended to the self renewal and maintenance of multipotency in adult stem cell (ASC) populations. This profile has arisen mainly from reports utilising reverse transcription-polymerase chain reaction (RT-PCR) based methodologies and has since come under scrutiny following the discovery that many developmental genes have multiple pseudogenes associated with them. Six known pseudogenes exist for Oct4, all of which exhibit very high sequence homology (three >97%), and for this reason the generation of artefacts may have contributed to false identification of Oct4 in somatic cell populations. While ASC lack a molecular blueprint of transcription factors proposed to be involved with 'stemness' as described for ES cells, it is not unreasonable to assume that similar gene patterns may exist. The focus of this work was to corroborate reports that Oct4 is involved in the regulation of ASC self-renewal and differentiation, using a combination of methodologies to rule out pseudogene interference. Haematopoietic stem cells (HSC) derived from human umbilical cord blood (UCB) and various differentiated cell lines underwent RT-PCR, product sequencing and transfection studies using an Oct4 promoter-driven reporter. In summary, only the positive control expressed Oct4, with all other cell types expressing a variety of Oct4 pseudogenes. Somatic cells were incapable of utilising an exogenous Oct4 promoter construct, leading to the conclusion that Oct4 does not appear involved in the multipotency of human HSC from UCB.

## Introduction

Octamer binding protein 4 (Oct4, also known as Pou5f1 and Oct3) is the transcription factor most associated with and critical for maintenance of totipotency in blastomeres and pluripotency in the inner cell mass of developing mammalian embryos [1,2]. Belonging to the POU domain family of transcription factors [2], Oct4 mediates activation or repression of target genes involved in stem cell differentiation, either as a dimeric trans-activator of gene expression or synergistically with other transcription factors such as Sox2 [3]. Until recently, Oct4 was thought to be expressed exclusively in embryonic stem (ES) cells and primordial germ cells. Several recent studies have proposed however, that Oct4 may also regulate adult stem cell multipotency, with expression detected in a variety of tissues including: bone marrow, peripheral blood and umbilical cord blood (UCB) derived cells [4-9], human progenitor-like cells from liver [10], skin epidermis [11] and hair follicles [12]. While the prospect of such genes being involved in somatic stem cell self-renewal and differentiation is appealing, caution is necessary following the discovery that some developmental genes have multiple pseudogenes [13].

Mammalian genomes contain many gene-like sequences which appear similar to functional genes, but which contain defects that either prevent transcription or generate non-functional protein transcripts. A duplicated pseudogene often lacks regulatory regions [14] and may arise from gene duplication or unequal crossing-over during meiosis and can retain some intron/exon boundaries observed in the parental gene. Alternatively, processed pseudogenes or retro-transposons represent the mRNA form of the gene rather than the DNA encoded sequence and lack both intron and promoter sequences. The belief is that mRNA is converted into DNA via a reverse transcriptase event and randomly inserted back into the genome [15]. If insertion places the pseudogene under the influence of a nearby active promoter, it may consequently be expressed. As with duplicated pseudogenes, accumulation of DNA mutations prevents coding of functional proteins. Nonetheless, evidence suggests that some pseudogene transcripts may play regulatory roles in expression of the parental gene, potentially by an RNA interference-like mechanism [16-19]. Hence some pseudogenes are transcribed and as such may generate false positives in hybridisation or amplification experiments.

Experimental prudence is therefore advisable due to the high sequence homology between developmental genes and their pseudogene transcripts. At present, *Oct4 *has six known pseudogenes, all having a minimum of 84.7% sequence identity with genuine *Oct4*, three of which have >97% [19]. Similarly for other developmentally important genes such as *Nanog *and *Stella*, numerous pseudogenes with coding regions containing >90% sequence conservation have been identified [13]. Many studies to date have relied on RT-PCR alone as method of detecting *Oct4 *in adult stem cells, with few including other molecular techniques. In the present study, our aims were to corroborate reports that developmental genes such as *Oct4 *are involved in regulation of multipotency in human adult progenitor cells, using a combination of methodologies. Haematopoietic stem cells derived from human UCB underwent RT-PCR analysis using primers reported to preclude pseudogene amplification, followed by product sequencing. Additionally, transfection studies utilising a human Oct4 promoter construct [20], determined whether cells were capable of expressing the factors necessary for genuine *Oct4 *gene expression.

## Results

**Figure 1:**
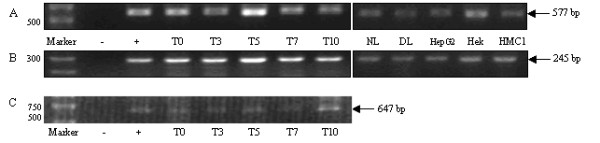
**RT-PCR analysis of Oct4**. A) Expression of Oct4 (Primer set 1, 577 bp) in freshly isolated c-kit^+ ^haematopoietic cells (T0) and following culture (T3--T10); EC 2102Ep cells (+ control); water (- control); normal hepatocytes (NL); diseased hepatocytes (DL); HepG2 cells; Hek293T cells (Hek); human mast cell line (HMC1). B) β-actin controls (245 bp). C) Expression of Oct4 (Primer set 2, 647 bp) in c-kit^+ ^cells (T0-T10); EC 2102Ep (+ control); water (-).

Following recent reports of *Oct4 *expression in various adult stem cell populations [4-12], we examined whether human c-kit^+ ^HSCs from UCB were also capable of expressing this early developmental gene, using RT-PCR, sequencing and transfection studies. Initial RT-PCR results using Oct4 primer set 1 [21], suggested that both freshly isolated and cultured c-kit^+ ^cells expressed *Oct4 *(figure 1A). At the time this work was undertaken however, two reports highlighted evidence that ES cell genes involved in pluripotency appeared to have a high proportion of pseudogene expression associated with them [13,22]. With this in mind, PCR was repeated using differentiated cell types and cell lines not anticipated to express *Oct4*. The predicted *Oct4 *transcript size of 577 base pairs (bp) was generated in normal and diseased hepatocytes, as well as in cell lines HepG2, Hek293T and HMC-1 (figure 1A). These results suggested the possibility of pseudogene amplification. Using NCBI BLAST software, various published Oct4 primer sequences [10,11,21,23,24] were checked and found to align with at least one known pseudogene, in addition to the genuine *Oct4 *transcript (data not shown). With *Oct4 *having 6 highly homologous retro-transposons already identified (three of which having >97% sequence homology), designing primers capable of recognising only genuine *Oct4 *transcripts proved to be a challenge. Subsequently, primers described to preclude the amplification of *Oct4 *pseudogenes were published [25] and RT-PCR analysis repeated. A band of predicted size (647 bp) was generated using primer set 2 in the positive control (embryonic carcinoma (EC) 2102Ep cells) and c-kit^+ ^progenitor cells (figure 1C). As before however, the cell lines HepG2, Hek293T and HMC-1 also appeared to amplify an *Oct4 *transcript (data not shown).

**Figure 2:**
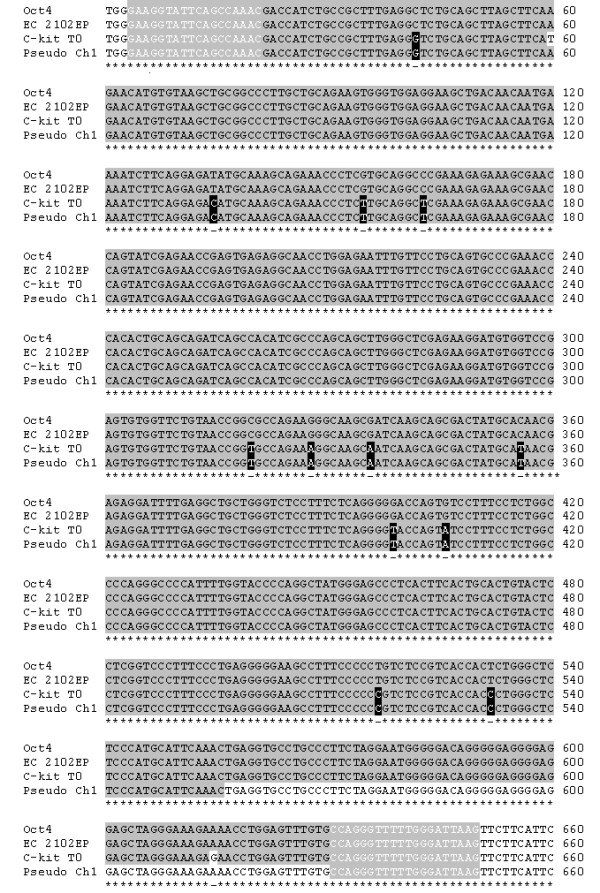
**Oct4 sequence alignments**. Alignment of Oct4 mRNA and pseudogene sequence from chromosome 1, with putative Oct4 RT-PCR sequenced products from freshly isolated c-kit^+ ^cells (T0) and EC 2102Ep cells (+ control). The solid grey background indicates sequence homology, with base pair mismatches highlighted by white background. Black boxes indicate homology between c-kit^+ ^and pseudogene sequences and disparity with Oct4. White letters on grey background denote primer positions.

Sequencing of the PCR products amplified by primer set 2 revealed which cell types were capable of expressing genuine Oct 4. A summary of the sequencing data can be found in table 1 and shows that only the positive control cells (EC 2102Ep) aligned 100% against the NCBI BLAST sequence of Oct4 (figure 2 and table 1). Results obtained for both freshly isolated and cultured c-kit^+ ^cells illustrated the high level of homology between the c-kit^+ ^cell transcripts and the *Oct4 *sequence (figure 2, 97.6%), however ~14 base pair mismatches indicated the probability of pseudogene amplification. Comparison of PCR-amplified cDNA from c-kit^+ ^cells with known *Oct4 *retro-transposon sequences confirmed that at least three pseudogenes were being expressed in the HSCs (table 1). These pseudogene alignments ranged from 99.5 - 99.8% sequence homology and were found on chromosomes 1, 10 and 12. *Oct4 *products generated in cell lines HepG2 and Hek293T also aligned with pseudogenes from chromosome 1 and 12 with 99% and 99.5% homology respectively (table 1).

**Table 1:** Summary of Oct4 sequencing data.

	Oct4 Chromosome 6	Pseudogene Chromosome 1	Pseudogene Chromosome 10	Pseudogene Chromosome 12
C-kit+ T0	No	Yes	Yes	-

C-Kit+ T7	No	-	Yes	Yes

EC 2102Ep	Yes	-	-	-

Hek293T	No	-	-	Yes

HepG2	No	Yes	-	-

Expression of Oct4 was observed in EC 2102Ep cells (+ control). All other cells expressed a variety of Oct4 pseudogene transcripts derived from chromosomes 1, 10 and 12. C-kit^+ ^cells (T0) n = 7, c-kit^+ ^cells (T7) n = 4, all other cell types n = 3.

**Figure 3:**
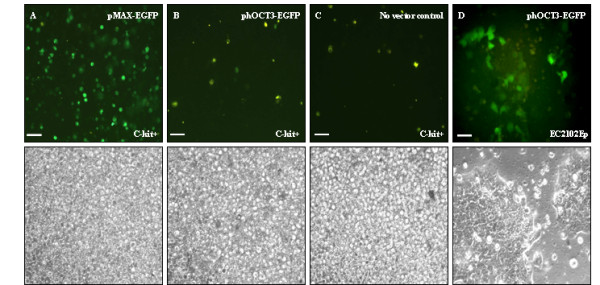
**Transfection studies on freshly isolated c-kit^+ ^cells**. Freshly isolated c-kit^+ ^cells transfected with: A) positive control vector pMAX-EGFP; B) phOCT3-EGFP; C) No vector negative control; D) EC 2102Ep cells transfected with phOCT3-EGFP. Lower panel represents phase contrast images of the above GFP images. Original magnification 100× (A, B, C) and 200× (D). Scale bar 50 μM.

Transfection studies were used as an alternative means to determine whether c-kit^+ ^progenitor cells expressed the transcription factors necessary to activate *Oct4 *gene expression. Freshly isolated c-kit^+ ^cells were rested overnight in expansion media before electroporation. At 48 h post-transfection, the only progenitor cell samples expressing GFP were the two positive controls, pMAX-EGFP (figure 3A) and pC1-EGFP (data not shown). C-kit^+ ^cells transfected with an Oct4 promoter driven construct phOCT3-EGFP1, were negative for GFP expression (figure 3B). The low level of non specific auto-fluorescence seen for c-kit^+ ^cells transfected with phOCT3-EGFP1 was comparable to the no vector negative control (figure 3C). Although comparison of fluorescence and phase contrast light microscopy illustrated the low level of transfection efficiency with control vectors (approximately 30% for pMAX-EGFP), this did confirm c-kit^+ ^progenitor cells were capable of expressing exogenous GFP (figure 3A). Similar results were observed with cultured c-kit^+ ^cells (transfected 11-14 days expansion), with the difference that fewer cells appeared to express GFP in both controls (data not shown). Transfection of the cell lines HepG2 and Hek293T generated similar results to those for c-kit^+ ^cells, both with pEGFP-C1 vector and phOCT3-EGFP1, in that none appeared capable of Oct4 driven GFP expression (data not shown). The only cell type to exhibit Oct4 promoter driven fluorescence was the cell line EC 2102Ep (figure 3D).

## Discussion

At present the molecular mechanisms involved in adult stem cell pluripotency and self renewal remain largely unknown. Furthermore, they appear to lack a blueprint of transcription factors proposed to be involved with 'stemness', as described for ES cells [26]. It is not unreasonable to assume however, that similar gene patterns may exist for adult stem cells. The focus of this work was to ascertain whether human adult stem cells, specifically c-kit isolated cells from UCB were capable of expressing transcription factors described for ES cell pluripotency. The impetus for the study arose over controversy following the number of recent reports suggesting genes such as *Oct4 *play a role in regulation of adult stem cell multipotency [4-12] and the proposed pre-disposition of developmentally active genes to retro-transposition events [13].

From our initial results, it became clear that reliance on RT-PCR technology was highly dependent on the specificity of primer sequences and was an unreliable method of analysing ES cell pluripotency genes in adult stem cells. Amplification of 'Oct4' transcripts in differentiated cell types such as hepatocytes and various cell lines (together with c-kit^+ ^progenitor cell populations), revealed the difficulty in designing primers capable of recognising only genuine *Oct4*. On examination of several published primer sequences, all aligned with *Oct4 *pseudogene sequences in addition to genuine *Oct4*, as determined using the NCBI BLAST alignment tool [10,11,21,23,24]. This raised the possibility that artefacts generated by *Oct4 *pseudogenes may have contributed to false identification of *Oct4 *and our current knowledge of adult stem cell gene expression. Recently, Liedtke *et al *[27] approached this problem by alignment of *Oct4 *to all its known pseudogene sequences with design of exact primers incorporating a polymorphism unique to genuine *Oct4*. They demonstrated that their primers could discriminate between cDNA and genomic DNA. Furthermore, in accordance with our data, Liedtke et al [27] showed that *Oct4 *was not expressed in human cord blood mononuclear (MN) cells or peripheral blood MN cells, contradicting other recent reports [5,7,28].

In both ES and EC cell types, pseudogene expression appears less of a contaminating factor in such cell populations. This may be attributed to events which occur during early development, whereby genes which are not involved in embryonic patterning are silenced by promoter methylation. Genes such as *Oct4 *have an 'anti-silencing' promoter which prevents methylation during this stage of embryonic development [29]. Hence, lack of pseudogene expression in these cells may likely be due to such generic gene silencing. Our sequencing results however, suggest that neither freshly isolated nor cultured c-kit^+ ^progenitor cells (nor cell lines HepG2, Hek293T and HMC1) undergo such tight regulation of *Oct4 *pseudogene expression, whilst they also appear to be incapable of genuine *Oct4 *transcription. The reason why the frequency of retro-transposition events in ES cell-specific genes far exceeds those of non-ES cells is not known [13]. Interestingly, this may have particular significance with regard to recent findings by Elliman et al [30] who have detected pseudogene expression of *Dppa3/Stella *in human adult tissue types including bone marrow, peripheral blood, pancreas, adrenal and thyroid gland. This supports the fact that developmental pseudogene expression in differentiated tissues is more widespread than previously thought and reiterates the need for caution regarding use of such genes as markers of adult stem cell pluripotency.

Our subsequent strategy was to select published primers designed to specifically amplify genuine *Oct4 *gene expression [25]. Unfortunately, our sequencing results confirmed that in non ES cell samples, these too detected pseudogene expression. On inspection of where these primers aligned on genomic DNA from chromosome 6, the reason why they may have failed to preclude pseudogene amplification became apparent. The anti-sense primer sequence taken from Pickering *et al *[25] had not incorporated an additional guanine base present in the *Oct4 *sequence obtained from NCBI, accession number NC_000006. Subsequent 'BLASTing' with the new primer sequence generated alignments to several pseudogene transcripts, thus providing an explanation for our unexpected RT-PCR false positives. (NB: the *Oct4 *DNA sequence used to identify primer sequences was NCBI version GI:51511722 which replaced version GI:42406225 on 24 august 2004). Therefore, it is possible that the previous DNA sequence had omitted the guanine base identified and may have influenced original primer design. Our sequencing results revealed that only the EC cell positive control expressed the genuine *Oct4 *transcript with 100% sequence homology to the 647 bp product. All the other cell types examined expressed a selection of the various *Oct4 *pseudogene mRNAs found on chromosomes 1,10 and 12, all which have >97% homology to genuine *Oct4 *[19].

Recently, several reports have proposed that pseudogene transcripts may act as regulators of protein synthesis and mRNA stability, particularly during carcinogenesis [31,18,19]. Kandouz et al [18] revealed that a *Connexin43 *pseudogene [ψCx43] plays a functional role in some tumour cells. The protein Connexin43, involved in intercellular communication is often deregulated in many cancers. Interestingly, in some cancer cells ψCx43 becomes translated into a functional protein, playing a role in growth inhibition. This appears to contradict the common concept that pseudogenes represent a version of a gene mutated to inactivity. If however, the mutation is within a regulatory region which prevents transcription, perhaps under certain cellular conditions the pseudogene may be expressed. This putative role of *Oct4 *pseudogene transcripts in regulation of the parent gene has been proposed by Suo et al [19] who identified two pseudogenes transcribed in conjunction with the apparent genuine *Oct4 *in HepG2, MCF-7 and Hela cell lines. Nevertheless, this is speculative and recent work by Mueller et al [32], using a variety of techniques and in excess of 30 somatic tumour cell lines, have concluded that functional Oct4 is not expressed. As with our results, they also demonstrated that the only cells to exhibit genuine *Oct4 *expression were EC cell lines [32].

Although sequencing data in the present study revealed no genuine *Oct4 *expression in c-kit^+ ^cells, we wished to investigate whether these cells were capable of utilising an exogenous Oct4 promoter construct. The reasoning was that if levels of pseudogene transcripts were relatively high in comparison to their genuine *Oct4 *counterparts, the pseudogenes may have been preferentially amplified. Our results corroborated the sequencing data in that the only cells able to express genuine *Oct4 *were the EC 2102Ep cells, with no GFP observed in c-kit^+ ^HSCs or cell lines HepG2 and Hek203T. In summary our results suggest that *Oct4 *does not appear to be involved in adult stem cell multipotency, in human UCB progenitor cells. In support of our findings, work by Lengner *et al *[33] have shown recently the same appears true for a variety of mouse somatic stem cells, using *Oct4 *gene ablation. They show that *Oct4 *is dispensable for self-renewal in intestinal epithelium, bone marrow mesenchymal stem cells and HSC, hair follicle, brain and liver in contrast with previous findings utilising mainly RT-PCR analysis.

In conclusion, together with a number of recent reports [27,32,34,35] on the controversy surrounding the role of Oct4 in ASCs, our study has underlined the necessity of utilising more than one approach to identify embryonic genes involved in pluripotency before hypothesising their involvement in ASC multipotency. We were unable to substantiate recent reports that Oct4 is involved in the self-renewal of somatic stem cell populations, reiterating the need for caution in the interpretation of results, especially RNA derived when investigating genes prone to high level pseudogene expression.

## Materials and methods

### Cell Culture

#### Human Adult Stem Cells

UCB was collected following local ethics committee approved informed consent from women undergoing full-term elective caesarean sections at Birmingham Women's Hospital. Freshly isolated blood was treated with 10% sodium citrate solution to prevent clotting and the mononuclear fraction of cells isolated using a sucrose density gradient (Ficoll-Paque™, UK). Contaminating red blood cells were depleted by ammonium chloride treatment, followed by purification of a c-kit^+ ^progenitor cell population using positive immuno-selection (EasySep™ Technique, StemCell Technologies Inc, UK: CD117/c-kit R-PE IgG1κ, clone YB5.B8, BD PharMingen Biosciences, UK). The percentage yield and purity of isolated cells was ascertained by flow cytometry: the mononuclear cell fraction consisted of 4% c-kit^+ ^cells, which following isolation using EasySep™, gave a c-kit^+ ^population of 89% purity (data not shown). Progenitor cells were maintained in suspension, in Stem Span™ serum free media with addition of 100 ng/ml stem cell factor (SCF), 10 ng/ml flt-3 ligand, 10 ng/ml thrombopoietin and 2 mM L-glutamine (Stem Cell Technologies Inc, UK). Cells were plated at a density of ~2 × 10^5 ^cell/ml and media changed every 4-5 days.

#### Human Cell Lines

The embryonic carcinoma (EC) cell line 2102Ep (kindly provided by Prof PW Andrews, University of Sheffield) was cultured on plastic, in DMEM supplemented with 10% foetal calf serum (FCS) and 2 mM L-glutamine. The human derived cell lines HepG2, human embryonic kidney Hek293T and human mast cell HMC-1 were routinely cultured in DMEM supplemented with 10% FCS, 2 mM L-glutamine, 1 mM sodium pyruvate, 1% nonessential amino acids and 1% penicillin/streptomycin. Media was changed every other day and all cells were maintained at 37°C in a humidified atmosphere of 5% CO_2 _in air.

### RT-PCR Analysis of Gene Expression

Total RNA was extracted from freshly isolated and cultured c-kit^+ ^UCB cells and cell line pellets using Rnazol (BioGenesis, UK, 1 ml/~10^8 ^cells) and chloroform (200 μl/~10^8 ^cells). Following agitation, incubation on ice and centrifugation (6000 *g*, 15 min), the aqueous phase was removed and added to an equal volume of isopropanol and stored overnight at 4°C. Samples were centrifuged (6000 *g*, 30 min), supernatant discarded and the pellet washed in 75% (v/v) ethanol. The final RNA pellet was then resuspended in 10 μl PCR water (Sigma-Aldrich Co. UK) and contaminating genomic DNA removed following treatment with DnaseI (Amersham Biosciences, UK). RNA samples were stored at -80°C.

cDNA was generated from mRNA under standard conditions using 100 μM random hexamers, 10 mM dNTPs (Amersham Biosciences) 50 mM MgCl_2_, 10× PCR buffer (Invitrogen), 50 mM Rnasin^® ^ribonuclease inhibitor (Promega, UK) and superscript II. cDNA samples were stored at -20°C. The polymerase chain reaction contained 10× PCR buffer, 50 mM MgCl_2_, 10 mM of each dNTP, 25 μM primers, PCR water and Taq DNA polymerase. The PCR primers used for amplifying Oct4 were those reported by Henderson *et al *[21] (set 1) and Pickering *et al *[25] (set 2). Oct4 primer set 1: forward 5' CGACCATCTGCCGCTTTGAG 3', reverse 5' CCCCCTGTCCCCCATTCCTA 3'; Oct4 primer set 2: forward 5' GAAGGTATTCAGCCAAAC 3', reverse 5' CTTAATCCAAAAACCCTGG 3'. β-actin forward 5' CATCACCATTGGCAATGAGC 3', reverse 5' CGATCCACACGGAGTACTTG 3'. The PCR cycling parameters used for each primer set consisted of an initial cDNA denaturation of 3 min at 94°C, followed by a cycle of 1 min at 94°C, 1 min at annealing temperature and DNA extension for 1 min at 72°C. Generally, the number of amplification cycles was 30-35 maximum. A final extension step was performed at 72°C for 10 min and held at 4°C until analysis. All experiments included negative controls with no cDNA added. PCR products were resolved by 1% agarose gel electrophoresis and visualised using ethidium bromide staining. Oct4 RT-PCR products were confirmed by sequencing.

### DNA Sequencing

Amplified DNA was extracted from PCR gels using QIAEX II kit (as described by the manufacturer, Qiagen, UK). Purified DNA was sub-cloned into a pGEM^®^-T Easy Vector (Promega), followed by heat shock transformation of JM109 competent cells. Amplified vector was recovered using QIAGEN Maxiprep and DNA sequenced at a final concentration of 400 ng/sample via Plasmid to Profile, version 3 (Genomics Lab, The University of Birmingham). DNA analysis was performed using FinchTV version 1.4 (^© ^Geospiza Inc), GeneDoc (^© ^1996-2000 Karl Nicholas) software programs and NCBI BLAST.

### Cell Transfections

Transfection of adherent cell lines was performed using Lipofectamine™ 2000 (Invitrogen, UK) and for primary progenitor cells cultured in suspension, cells underwent transfection by Amaxa electroporation (program U-08, Amaxa Biosystems, UK), both according to manufacturer guidelines. Adherent cells were grown in 25 cm^2 ^flasks and harvested by trypsinisation at approximately 80 - 95% confluence following 24 hr culture in antibiotic free media. Progenitor cells were transfected within 24 h of isolation and following culture up to day 14. Due to low cell numbers, progenitor cells were transfected at the lower end of the recommended range at a final concentration of 2 × 10^5 ^cells/100 μl. The 8068 bp Oct4 vector [20] consisted of the human Oct4 promoter region cloned into an enhanced GFP expressing plasmid (kindly donated by Dr W Cui, The Roslin Institute, Edinburgh). Positive control vectors pEGFP-C1 (4731 bp, GenBank, UK) and pMax-GFP (3486 bp, Amaxa Biosystems), were used to test transfection efficiency for both systems.

## Competing interests

The authors declare that they have no competing interests.

## Authors' contributions

ZR participated in the conception and design of the study, carried out all experimental work, collection of data, data analysis and manuscript writing.

AJS participated in the conception and design of the study and final approval of the manuscript.
